# Exploring influencing factors of chronic obstructive pulmonary disease based on elastic net and Bayesian network

**DOI:** 10.1038/s41598-022-11125-8

**Published:** 2022-05-09

**Authors:** Dichen Quan, Jiahui Ren, Hao Ren, Liqin Linghu, Xuchun Wang, Meichen Li, Yuchao Qiao, Zeping Ren, Lixia Qiu

**Affiliations:** 1 Department of Health Statistics, School of Public Health, Shanxi Medical University, Taiyuan, Shanxi China; 2 Shanxi Centre for Disease Control and Prevention, Taiyuan, 030012 Shanxi China

**Keywords:** Chronic obstructive pulmonary disease, Epidemiology

## Abstract

This study aimed to construct Bayesian networks (BNs) to analyze the network relationships between COPD and its influencing factors, and the strength of each factor's influence on COPD was reflected through network reasoning. Elastic Net and Max-Min Hill-Climbing (MMHC) algorithm were adopted to screen the variables on the surveillance data of COPD among residents in Shanxi Province, China from 2014 to 2015, and construct BNs respectively. 10 variables finally entered the model after screening by Elastic Net. The BNs constructed by MMHC showed that smoking status, household air pollution, family history, cough, air hunger or dyspnea were directly related to COPD, and Gender was indirectly linked to COPD through smoking status. Moreover, smoking status, household air pollution and family history were the parent nodes of COPD, and cough, air hunger or dyspnea represented the child nodes of COPD. In other words, smoking status, household air pollution and family history were related to the occurrence of COPD, and COPD would make patients’ cough, air hunger or dyspnea worse. Generally speaking, BNs could reveal the complex network linkages between COPD and its relevant factors well, making it more convenient to carry out targeted prevention and control of COPD.

## Introduction

Chronic obstructive pulmonary disease (COPD) represents a common disease characterized by persistent respiratory symptoms and airflow limitation. Its clinical manifestations comprise cough, expectoration, chest tightness, air hunger or dyspnea. In severe cases, it would progress into respiratory failure and pulmonary heart disease, which can have a significant impact on patients’ quality of life. It has emerged as the fifth largest economic burden worldwide^1,2^, especially in developing countries, where COPD owns high morbidity and mortality. One study has shown that about 1 million people die of COPD in China every year, accounting for 30% of COPD deaths globally^3^. Additionally, COPD constitutes the third and fourth leading cause of death in China’s rural and urban areas, respectively^4^. Obviously, COPD has become an important public health problem. To comprehensively analyze its influencing factors and the complex relationship between them, and thus to reduce occurrence is all the more important.

Previously, studies on COPD generally explored the influencing factors by logistic regression, which required those factors to be inter-independent, and reflected the correlations between factors and COPD based on odds ratio. For example, in 2018, Wang Chen^5^ utilized logistic regression to detect risk factors of COPD, suggesting that sex, age, years of smoking, and severe exposure to PM2.5 were significantly associated with COPD. However, in clinical research, correlation often exist in influencing factors of disease. Therefore, it fails to meet the prerequisite of independent variables. In addition, logistic regression is unable to reveal direct or indirect influencing factors of COPD^6^.

Bayesian networks (BNs), proposed by Pearl Judea in 1987, have been widely used^7^. Without strict statistical hypothesis^8^, BNs constitute directed acyclic graph (DAG), reflecting potential relationship among influencing factors, and conditional probability distribution table (CPT), which demonstrates the correlation intensity among factors^9^. As such, it allows for complex networks between disease and its influencing factors, overcoming the limitation of traditional logistic regression^10^. Moreover, with the information of known nodes, BNs can infer the probability of unknown nodes, flexibly showing the impact of relevant risk factors on COPD^11,12^.

BNs learning refers to obtaining a complete Bayesian network by existing information. The construction method consists of parameter learning and structure learning^13^. The former one assumes that the network structure is known, then determining the parameters in the network. This article focuses on structure learning, a more commonly-used method, which could be divided into score-based search^14^ and constraint-based algorithm^15^.

The essence of score-based search is to find out the BNs model whose score function reaches the maximum. Nonetheless, it’s hard^16^ to obtain an optimal network structure under large structure space. The constraint-based algorithm enjoys a high learning efficiency and can obtain global optimal solution, but it also comes with some shortcomings. Firstly, independence of different nodes is sophisticated to judge, and the independence tests between nodes increase exponentially with the increase of nodes. Secondly, the results of high-order conditional independence test are unreliable. Due to their limitations, some scholars have proposed a hybrid algorithm, Max-Min Hill-Climbing (MMHC), a widely used hybrid algorithm, which includes two phases. In the first stage, it builds an undirected framework of BNs to reduce the search space by using constraint-based algorithms. Then the score-based search is used to add, delete and change the direction of edges in the constrained space, to find the network with the highest score^16^. Thus, MMHC hybrid algorithm combines the two algorithms skillfully and overcomes their shortcomings effectively^17^.

Undoubtedly, risk factors for COPD abound, but it’s improper to corporate all these factors into BNs. The networks would become extremely complicated and less accurate with more factors. So feature selection is of great necessity. Since Lasso regression does not take into account the correlation between features, it is not suitable for screening variables with multicollinearity. Ridge regression cannot select the model as well, on account of no prediction factor with zero actual coefficient. However, Elastic Net^18^ can combine the two methods and carry on the feature selection through cross-verification. By adopting Elastic Net, an ideal sparse model can be obtained and the influence of correlation between the observed variables can be compensated.

Hence, we intended to employ Elastic Net to filter factors, which had strong correlations with COPD, and then used MMHC to build BNs of COPD and selected factors, exploring potential relationships between COPD and these factors, so as to provide theoretical guidance for prevention and reduction of COPD.

## Results

### Characteristics of the study population

**Figure 1:**
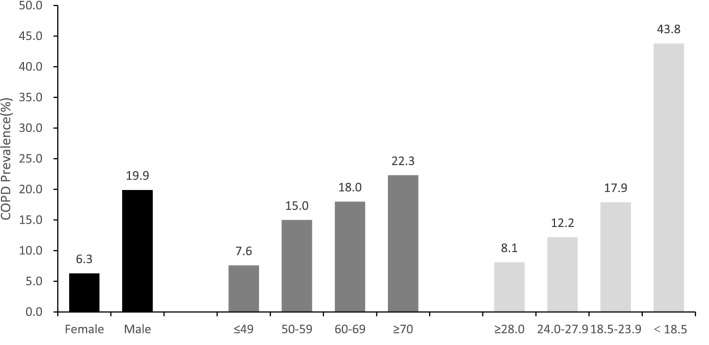
The prevalence of COPD in different gender, age and BMI.

Among the 2424 initial study participants, 352 respondents with incomplete data were excluded, and 2072 were left for the analysis. Among them, 51.8% were men and 48.2% were women. 36.6% of the participants were aged between 40 and 49; 35.0% were between 50 and 59; 23.4% were between 60 and 69, and 5.0% were over 70 years old (As shown in Supplementary Table S1). The prevalence of COPD in this study represented 13.4% (19.9% in males and 6.3% in females). The prevalence of COPD increased with age, with the highest rate being 22.3% in those older than 70 years, as shown in Fig. 1.

**Table 1:** Selected variables and their regression coefficients.

Variable	Coefficient	Variable	Coefficient
Gender (x_1_)	0.24111383	Air hunger or dyspnea (x_8_)	0.07155523
Age (x_2_)	− 0.07398471	Respiratory disease (x_10_)	0.16883457
BMI (x_4_)	0.074471365	Family history (x_13_)	0.04980561
Cough (x_6_)	0.22663873	Smoking status (x_14_)	0.22661544
Expectoration (x_7_)	0.13507333	Household air pollution (x_15_)	0.08082141

### Screening of variables associated with COPD by Elastic Net

16 risk factors related to COPD were included in the Elastic Net model, and the key parameter values (λ = 0.18595, α = 0.12) for optimizing the model performance were selected by a ten-fold cross-validation method. In the end, the coefficients of influencing factors not closely related to COPD would be compressed to 0 and eliminated, and the final 10 variables were obtained, as shown in Table 1. This method was used to determine the factors that had strong correlations with COPD, thus simplifying the structure of BNs.

### Bayesian networks model of COPD

**Figure 2:**
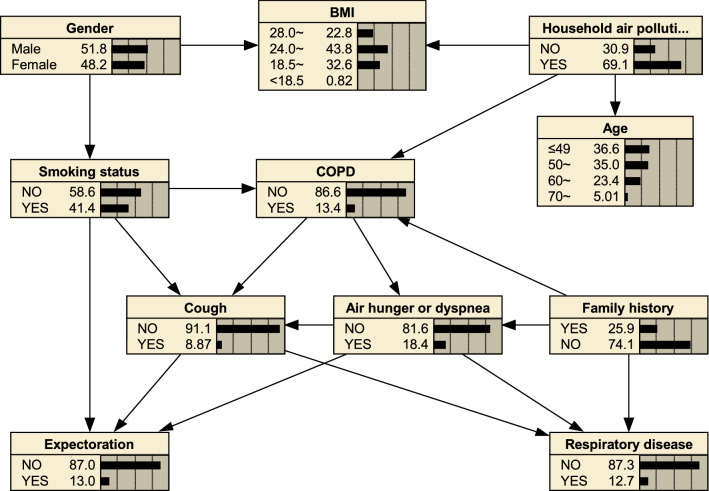
MMHC algorithm to construct COPD Bayesian networks and prior probability.

As shown in Fig. 2, a BNs model with 11 nodes and 18 directed edges was constructed. The directed edges represented probabilistic dependence between connected nodes. The numbers in the figure represented the prior probability of each node. For example, the prior probability of COPD was 0.134, that is, P(COPD) = 0.134. The results showed that smoking status, household air pollution, family history, cough, air hunger or dyspnea were directly related to COPD. Among them, smoking status, household air pollution, and family history constituted the parent nodes of COPD, that is, they were related to the occurrence of COPD. Cough, air hunger or dyspnea were child nodes of COPD. Namely, COPD was related to the occurrence of Cough, Air hunger or dyspnea.

### Reasoning model of COPD

BNs can infer the probability of an unknown node based on the state of known nodes, and make COPD risk prediction possible. If an individual smokes, the probability of suffering from COPD is 0.215, that is, P(COPD | Smoking status) = 0.215, as shown in Supplementary Fig. S1; if this person also used wood, animal feces or coal in the past 6 months or more to cook or heat, the probability of suffering from COPD will rise to 0.246, that is, P(COPD | Smoking status, Household air pollution) = 0.246, as shown in Supplementary Fig. S2; if this person has a family history of respiratory disease at the same time, then the possibility of suffering from COPD goes up to 0.280, that is, P(COPD | Smoking status, Household air pollution, Family history) = 0.280, as shown in Supplementary Fig. S3. Similarly, when a person suffers from COPD, the probability of Cough rises from 0.0887 to 0.201, and the probability of Air hunger or dyspnea increases from 0.184 to 0.289, as shown in Supplementary Fig. S4.

## Discussion

As the prevalence and morbidity continue to rise, COPD has become an important public health issue. Globally, it is the main cause of disability among elderly population and has become the fifth largest burden on the global economy^2^. This study showed that the prevalence of COPD in Shanxi Province, China was 13.4% in 2014, which was comparable to the national COPD prevalence of 13.6%. However, over the past decade, the prevalence of COPD among residents over 40 in China has increased from 8.2% in 2002^19^ to 13.7% in 2012^5^. This indicated that sufficient attention should be paid to the prevention and treatment of COPD.

The BNs constructed by the MMHC algorithm can explore the complex network connections between COPD and its influencing factors. The results of BNs model showed that smoking status, household air pollution, and family history were directly related to COPD, and gender was indirectly related to COPD through smoking. In addition, the BNs can also describe the relationship between other factors, such as family history, respiratory disease, air hunger or dyspnea, cough and expectoration, as shown in Fig. 2. Besides, CPT could show us how particular a risk factor causes an increased risk of developing COPD. Supplementary Table S2. illustrated the probability dependence between COPD and the three-parent nodes of smoking status, household air pollution, and family history. As we can see, if an individual had smoking status, household air pollution, family history at the same time, then this person was 28.0% likely to develop COPD, with P (COPD | smoking status, household air pollution, family history) = 0.280.

Smoking is currently recognized as the most important risk factor for COPD. The chemicals and fine particles produced during tobacco burning are the main cause of chronic bronchial inflammation and airway obstruction. One study^20^ found smokers were 2.46 times more likely to develop COPD than non-smokers, after adjusting for other factors. In our study, the smoking rate of residents aged 40 years or older reached 41.4%, in which more than 70 percent of men smoke, reflecting the high prevalence of smoking. From the perspective of COPD prevention and control, tobacco control and non-exposure to tobacco smoke prove one of the most important interventions.

In 2016, WHO^21^ estimated that about 3.1 billion people in low- and middle-income countries still cooked with contaminated fuel, causing approximately 4.3 million premature deaths each year, equivalent to 7.7% of global deaths. It also accounted for one third of COPD deaths in low- and middle-income countries. It’s a common phenomenon for residents to use polluted fuel for cooking or heating. In this survey, household air pollution rate was as high as 69.1%. Long-term exposure to those harmful gases can easily lead to COPD, which is mainly caused by airway reaction. Therefore, residents should change the way of heating and cooking, and reduce harmful exposure.

Having a family history of respiratory diseases will increase the incidence of COPD, suggesting that genetic susceptibility may be strongly associated with COPD. At present, some studies have found that the polymorphisms of α-antitrypsin, matrix metalloprotein, tumor necrosis factor α, interleukin and other genes were related to the pathogenesis of COPD, but further research is needed to clarify^22–24^.

In this paper, BNs combined with Elastic Net were used to analyze the related influencing factors of COPD, and the conclusions were consistent with previous studies, suggesting that BNs allow for accurate detection of risk factors for one specific disease. Also, BNs can vividly describe the complex network risk mechanism of COPD, through which we can not only discover the risk factors of COPD, but also demonstrate the correlation between these risk factors. Although logistic regression, a model based on the condition of independence of each influencing factor, could detect the risk factors of COPD, it fails to explore what role a risk factor plays in the development of COPD.

Finally, there are some shortcomings in this paper. As the number of healthy subjects is far more than that of patients, data imbalance is a commonplace. BNs rely on prior information, but prior information imbalance may lead to a less efficacious and robust model, resulting in poor stability of the output. Therefore, the BNs may not give same output if tested on data with large sample size. Nevertheless, due to the low prevalence of COPD in the population, the issue of imbalance in data still persists to some extent, regardless of the increase in sample size. Next, we will apply BNs to diseases with higher prevalence, and seek to take some measures to handle data imbalance, such as Resampling. Additionally, since this is a cross-sectional study, BNs can only demonstrate the related factors of one disease, and further validation is needed for the causality. Besides, for the 2014–2015 COPD surveillance data, there were certain missing parameters or information, resulting in a limited amount of information. Our ongoing work is to collect more information, and to further investigate their relationship with COPD, so as to take more targeted measures in disease control and prevention.

## Methods

### Study participants

In this study, data were obtained from the COPD monitoring of residents from 2014 to 2015, which was carried out in Shanxi Province, China. After excluding missing data, 2072 valid cases were obtained. Based on multi-stage stratified random sampling, the survey was conducted among Chinese residents aged 40 years or older in Taiyuan, Datong, Linfen and Xinzhou of Shanxi Province. This survey included basic information (such as gender, age, cultural level), respiratory symptoms (such as cough, expectoration, air hunger or dyspnea), personal diseases (such as childhood respiratory, hypertension) and risk factors exposure (such as household air pollution, occupational exposure). These factors and their assignments were depicted in Supplementary Table S1. This study was approved by the China-Japan Friendship Hospital. Informed consent was signed by all study participants or their agents. All experiments and methods were performed in accordance with the relevant guidelines and regulations.

The eligibility criterion for this study was residents of Chinese nationals aged 40 years or older who had lived in the monitoring area for at least 6 months during the 12 months preceding the survey. The exclusion criteria were as follows: (1) residents living in functional areas, such as barracks, military, student dormitories, nursing homes; (2) residents with mental disorders or cognitive disorders, such as dementia, comprehension impairment, deafness; (3) tumor patients found and being treated; (4) residents with high paraplegia; (5) pregnant or lactating women.

### Quality control

To ensure the reliability and validity of data, strict measures had been taken in this study. The investigators received standardized professional training before the investigation, and conducted on-site investigation on the respondents after qualified inspection, and collected relevant data through questionnaires. On-site and remote quality control were implemented through synchronous recording. All measuring instruments were calibrated before measurement. All data were entered twice into a database and checked for errors or omissions.

### Elastic Net

Regularization is a technique for adding penalties to the objective function. This penalty controls the complexity of the model by reducing the value of regression coefficient. Elastic Net^18^ is a linear regression model with L1 and L2 norms as regularization matrix. Not only does it retain the characteristic to easily produce feature sparsity like Lasso method, but also inherits the stability of Ridge regression. Its algorithm formula is as follows.

1 $$    \hat{B} = {}_{\beta }^{{\arg \min }} \frac{1}{2}\left\| {{\text{Y}} - {\text{X}}\beta } \right\|_{2}^{2}  + \lambda \left( {\alpha \left\| B \right\|_{1}  + \frac{{\left( {{ \vdash }a} \right)}}{2}\left\| B \right\|_{2} } \right) $$

As we can see, λ represents the penalty coefficient and β is the regression coefficient. For the convex combination of regularized L1 and L2(the value of *ɑ* in the formula), the l1_ratio parameter is used for adjustment. The final value of the parameter is selected with the lowest model error by ten-fold cross-validation.

### Bayesian networks

A Bayesian network is a probability graph model, which can show the probability dependence intensity between factors. It is a directed acyclic graph based on probability theory and graph theory, which consists of nodes representing the variables U = {xi,…,${\text{x}}_{{\text{n}}}$} and the directed edges represent the relationship between variables^12^. If the edge from ${\text{x}}_{{\text{i}}}$ to ${\text{x}}_{{\text{j}}}$ exists^13^, then ${\text{x}}_{{\text{i}}}$ is the parent code of ${\text{x}}_{{\text{j}}} { }$ and ${\text{x}}_{{\text{j}}}$ is the child code of ${\text{x}}_{{\text{i}}}$. Each node can quantitatively describe the probability correlation between the node and its parent node through the attached conditional probability distribution table (CPT). In BNs, the formula for calculating the joint probability distribution function of all nodes is as follows.

2 $$ \begin{aligned} {\text{P}}\left( {{\text{x}}_{1} {,}{\text{x}}_{2} {,} \ldots {,}{\text{x}}_{{\text{n}}} } \right) & = {\text{P}}\left( {{\text{x}}_{1} } \right){\text{P}}_{{\left( {{\text{x}}_{2} |{\text{x}}_{1} } \right)}} \cdots {\text{P}}\left( {x_{{n|x_{1} ,x_{2,} \ldots ,x_{n - 1} }} } \right) \\ { } & = {\Pi }_{1}^{{\text{n}}} {\text{P}}\left( {{\text{x}}_{{\text{i}}} |{\uppi }\left( {{\text{x}}_{{\text{i}}} } \right)} \right) \\ \end{aligned} $$

${\uppi }\left( {{\text{x}}_{{\text{i}}} } \right){ }$ is the set of parent nodes of ${\text{x}}_{{\text{i}}}$,${{ \uppi }}\left( {{\text{x}}_{{\text{i}}} } \right) \subseteq \left( {{\text{x}}_{1} , \ldots ,{\text{x}}_{{{\text{i}} - 1}} } \right)$.When the value of ${\uppi }\left( {{\text{x}}_{{\text{i}}} } \right)$ is known, ${\text{x}}_{{\text{i}}}$ is conditionally independent of other variables in $\left( {{\text{x}}_{1} , \ldots ,{\text{x}}_{{{\text{i}} - 1}} } \right)$.

### MMHC

MMHC, as a BNs hybrid structure learning algorithm, is widely used. It consists of two stages. In the first stage, the Max-Min Parents and Children (MMPC) algorithm is employed. MMPC can determine the existence of edges without direction, from which the BNs can be constructed. The MMPC algorithm also includes two phases, the first phase starts from the empty set, and then variables are put into candidate parents and children (CPC) successively by using the max-min heuristic function. The first phase doesn’t end until all remaining nodes are independent of the target node, namely T; in the second phase, false positive nodes are deleted through the conditional independence test. For a subset of variables, called S (S⊆CPC), if ind (X, T|S) is true, X will be deleted from CPC.

In the second stage of the MMHC algorithm, the mountain climbing method is used to locally adjust the current model by adding, deleting and changing the direction of the edges, so as to get several undetermined models, and then calculate the score of each undetermined model to obtain the BNs with the highest score^15^.

### Definition

After the bronchodilation test, Participant whose ratio of forced expiratory volume in the first second (FEV1) to forced vital capacity (FVC) was less than 70%, was determined as patient with COPD. The age consisted of four groups: 40–49, 50–59, 60–69, ≥ 70; cultural level was divided into three levels: junior high school and below, senior high school, college diploma or above. Bodyweight was classified as underweight (BMI < 18.5 kg/m^2^); Normal body weight (BMI 18.5–23.9 kg/m^2^); Overweight (BMI 24.0–27.9 kg/m^2^); Obesity (BMI≥ 28.0 kg/m^2^). Current and former cigarette exposure were defined as smokers, while never exposed ones were defined as non-smokers. Household air pollution referred the use of wood, animal manure or coal for cooking or heating over the past 6 months or more. Exposure to dust or harmful gases at work (including farm work) was defined as occupational exposure. A family history of respiratory disease was defined as having one or both parents with asthma, chronic bronchitis or emphysema.

### Statistical analysis

Statistical description of influencing factors was performed using Microsoft Office Excel (version 2016). Elastic Net was employed to filter variables in Python software (version 3.7.0). The structure of BNs was constructed by the MMHC in R studio 4.0.5 (R Development Core Team), and the maximum likelihood method was used for parameter learning. The drawing of the BNs and reasoning models were realized by Netica (Norsys Software Corp., Vancouver, BC, Canada). Additionally, the maximum likelihood method was used to obtain the values for CPT.

## Supplementary Information

Supplementary Information 1.

Supplementary Information 2.

Supplementary Information 3.

Supplementary Information 4.

Supplementary Information 5.

## Data Availability

The data that support the findings of this study are available from the corresponding author upon reasonable request.
