# Supplementary figures and images for: Exploring influencing factors of chronic obstructive pulmonary disease based on elastic net and Bayesian network
Source: Sci Rep. 2022 May 9;12:7563. doi: 10.1038/s41598-022-11125-8 (PMC9085890; doi:10.1038/s41598-022-11125-8)

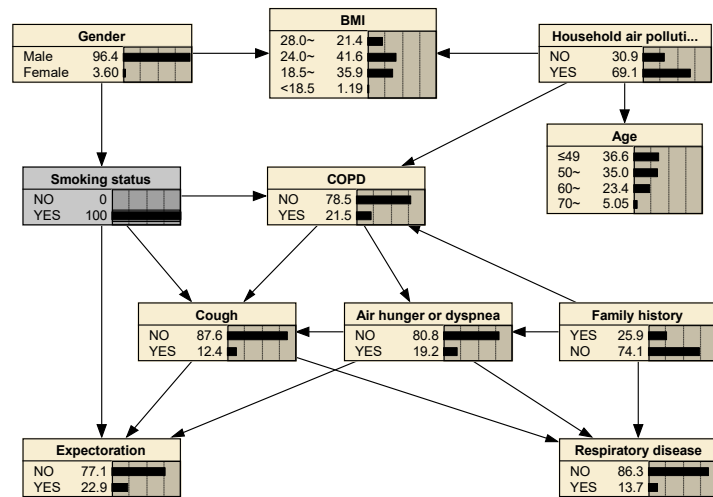

Supplement: Supplementary file 1 — Supplementary Information 1. [file 41598_2022_11125_MOESM1_ESM.pdf]

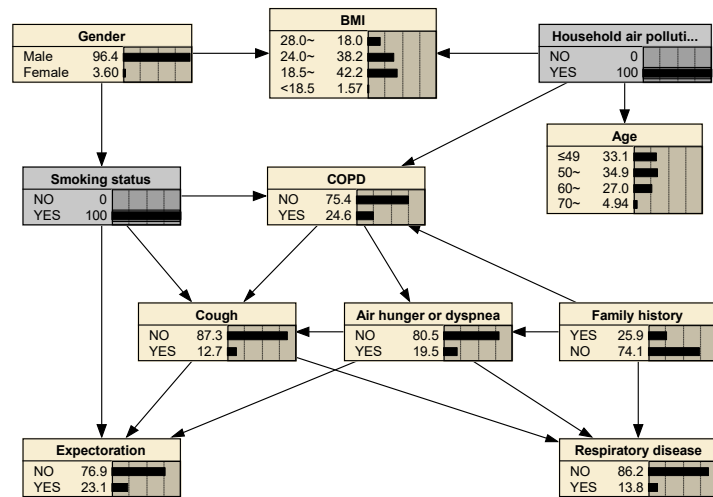

Supplement: Supplementary file 2 — Supplementary Information 2. [file 41598_2022_11125_MOESM2_ESM.pdf]

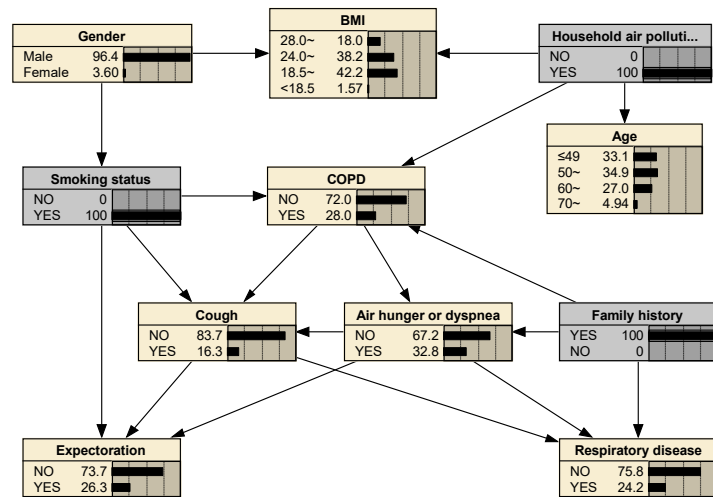

Supplement: Supplementary file 3 — Supplementary Information 3. [file 41598_2022_11125_MOESM3_ESM.pdf]

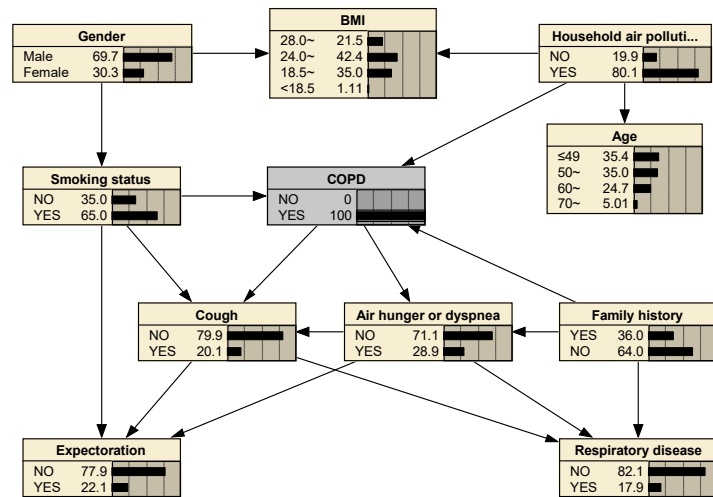

Supplement: Supplementary file 4 — Supplementary Information 4. [file 41598_2022_11125_MOESM4_ESM.pdf]
