# Supplementary material for: Exploring influencing factors of chronic obstructive pulmonary disease based on elastic net and Bayesian network
Source: Sci Rep. 2022 May 9;12:7563. doi: 10.1038/s41598-022-11125-8 (PMC9085890; doi:10.1038/s41598-022-11125-8)
Supplement: Supplementary file 5 — Supplementary Information 5. [file 41598_2022_11125_MOESM5_ESM.docx]

**Supplementary Tab.S1.** Factors and their assignments

| Factors | Assignments | Population | Percent（%） |
| --- | --- | --- | --- |
| COPD(Y) | YES=1 | 277 | 13.4 |
|  | NO=2 | 1795 | 86.6 |
| Gender($x_{1}$) | Male=1 | 1073 | 51.8 |
|  | Female=2 | 999 | 48.2 |
| Age($x_{2}$) | ≤49=1 | 759 | 36.6 |
|  | 50~=2 | 726 | 35.0 |
|  | 60~=3 | 484 | 23.4 |
|  | 70~=3 | 103 | 5.0 |
| Cultural level($x_{3}$) | Junior high and below=1 | 1557 | 75.1 |
|  | Senior high school=2 | 391 | 18.9 |
|  | College diploma or above=3 | 124 | 6.0 |
| BMI($x_{4}$) | <18.5=1 | 16 | 0.8 |
|  | 18.5∼=2 | 675 | 32.6 |
|  | 24.0∼=3 | 909 | 43.9 |
|  | 28.0∼=4 | 472 | 22.8 |
| COPD awareness ($x_{5}$) | YES=1 | 110 | 5.3 |
|  | NO=2 | 1962 | 94.7 |
| Cough ($x_{6}$) | YES=1 | 183 | 8.8 |
|  | NO=2 | 1889 | 91.2 |
| Expectoration ($x_{7}$) | YES=1 | 269 | 13.0 |
|  | NO=2 | 1803 | 87.0 |
| Air hunger or dyspnea($x_{8}$) | YES=1 | 381 | 18.4 |
|  | NO=2 | 1691 | 81.6 |
| Childhood respiratory infections ($x_{9}$) | YES=1 | 37 | 1.8 |
|  | NO=2 | 2035 | 98.2 |
| Respiratory disease ($x_{10}$) | YES=1 | 263 | 12.7 |
|  | NO=2 | 1809 | 87.3 |
| Cardiovascular and cerebrovascular diseases ($x_{11}$) | YES=1 | 141 | 6.8 |
|  | NO=2 | 1931 | 93.2 |
| Hypertension ($x_{12}$) | YES=1 | 449 | 21.7 |
|  | NO=2 | 1623 | 78.3 |
| Family history ($x_{13}$) | YES=1 | 536 | 25.9 |
|  | NO=2 | 1536 | 74.1 |
| Smoking status($x_{14}$) | YES=1 | 858 | 41.4 |
|  | NO=2 | 1214 | 58.6 |
| Household air pollution($x_{15}$) | YES=1 | 1432 | 69.1 |
|  | NO=2 | 640 | 30.9 |
| Occupational exposure ($x_{16}$) | YES=1 | 903 | 43.6 |
|  | NO=2 | 1169 | 56.4 |


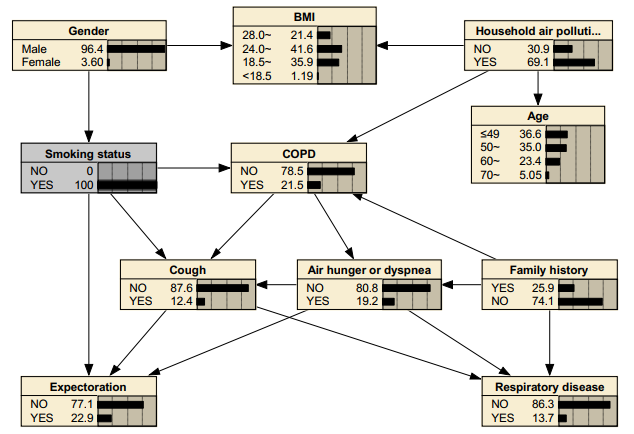


**Supplementary Fig.S1.** The Bayesian networksⅠunder known evidence variables. The figure was plotted using Netica (www.norsys.com).


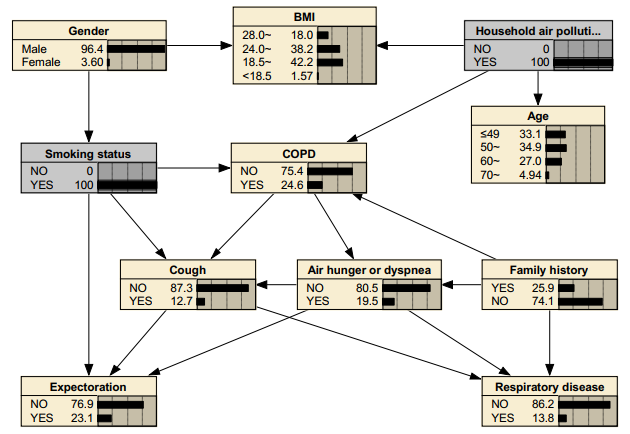


**Supplementary Fig.S2.** The Bayesian networks Ⅱ under known evidence variables. The figure was plotted using Netica (www.norsys.com).


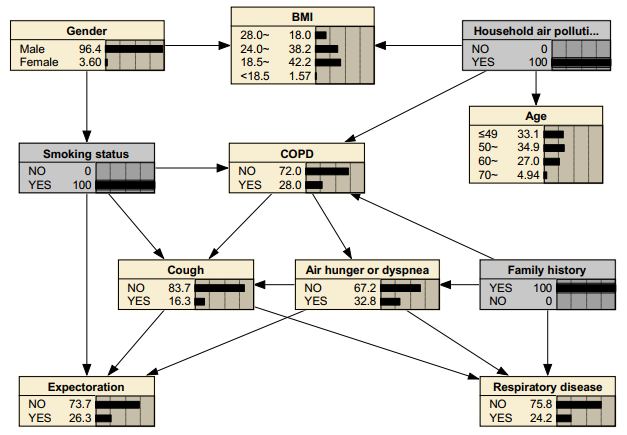


**Supplementary Fig.S3.** The Bayesian networks Ⅲ under known evidence variables. The figure was plotted using Netica (www.norsys.com).


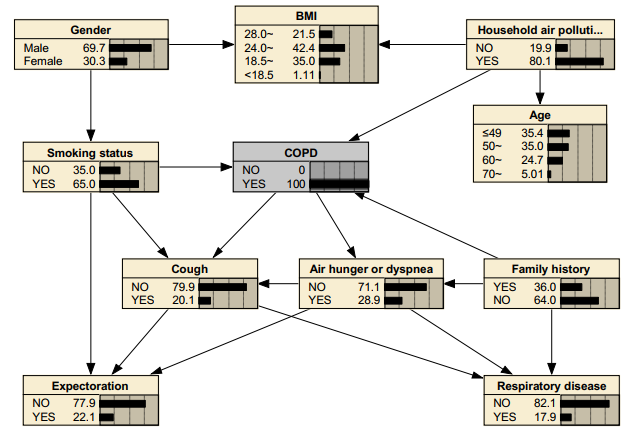


**Supplementary Fig.S4.** The Bayesian networks Ⅳ under known evidence variables. The figure was plotted using Netica (www.norsys.com).

**Supplementary Tab.S2.** Conditional probability table

| Family history | Smoking status | Household air pollution | COPD | |
| --- | --- | --- | --- | --- |
|  |  |  | NO | YES |
| Yes | No | No | 93.939 | 6.061 |
| Yes | No | Yes | 80.928 | 19.072 |
| Yes | Yes | No | 82.609 | 17.391 |
| Yes | Yes | Yes | 71.978 | 28.022 |
| No | No | No | 95.775 | 4.225 |
| No | No | Yes | 93.488 | 6.512 |
| No | Yes | No | 86.225 | 13.775 |
| No | Yes | Yes | 76.611 | 23.389 |

.
